# Development of a mutant aerosolized ACE2 that neutralizes SARS-CoV-2 *in vivo*

**DOI:** 10.1128/mbio.00768-24

**Published:** 2024-05-21

**Authors:** Daniel L. Kober, Marley C. Caballero Van Dyke, Jennifer L. Eitson, Ian N. Boys, Matthew B. McDougal, Daniel M. Rosenbaum, John W. Schoggins

**Affiliations:** 1 Department of Biochemistry, The University of Texas Southwestern Medical Center, Dallas, Texas, USA; 2 Department of Microbiology, The University of Texas Southwestern Medical Center, Dallas, Texas, USA; 3 Department of Biophysics, The University of Texas Southwestern Medical Center, Dallas, Texas, USA; Icahn School of Medicine at Mount Sinai, New York, New York, USA

**Keywords:** SARS-CoV-2, ACE2, receptors, receptor-ligand interaction, antiviral agents, mutational studies, protein structure-function

## Abstract

The rapid evolution of SARS-CoV-2 variants highlights the need for new therapies to prevent disease spread. SARS-CoV-2, like SARS-CoV-1, uses the human cell surface protein angiotensin-converting enzyme 2 (ACE2) as its native receptor. Here, we design and characterize a mutant ACE2 that enables rapid affinity purification of a dimeric protein by altering the active site to prevent autoproteolytic digestion of a C-terminal His_10_ epitope tag. In cultured cells, mutant ACE2 competitively inhibits lentiviral vectors pseudotyped with spikes from multiple SARS-CoV-2 variants and infectious SARS-CoV-2. Moreover, the protein can be nebulized and retains virus-binding properties. We developed a system for the delivery of aerosolized ACE2 to K18-hACE2 mice and demonstrated protection by our modified ACE2 when delivered as a prophylactic agent. These results show proof-of-concept for an aerosolized delivery method to evaluate anti-SARS-CoV-2 agents *in vivo* and suggest a new tool in the ongoing fight against SARS-CoV-2 and other ACE2-dependent viruses.

**IMPORTANCE:**

The rapid evolution of SARS-CoV-2 variants poses a challenge for immune recognition and antibody therapies. However, the virus is constrained by the requirement that it recognizes a human host receptor protein. A recombinant ACE2 could protect against SARS-CoV-2 infection by functioning as a soluble decoy receptor. We designed a mutant version of ACE2 with impaired catalytic activity to enable the purification of the protein using a single affinity purification step. This protein can be nebulized and retains the ability to bind the relevant domains from SARS-CoV-1 and SARS-CoV-2. Moreover, this protein inhibits viral infection against a panel of coronaviruses in cells. Finally, we developed an aerosolized delivery system for animal studies and show the modified ACE2 offers protection in an animal model of COVID-19. These results show proof-of-concept for an aerosolized delivery method to evaluate anti-SARS-CoV-2 agents *in vivo* and suggest a new tool in the ongoing fight against SARS-CoV-2.

## INTRODUCTION

The severe acute respiratory syndrome coronavirus 2 (SARS-CoV-2) causes coronavirus-induced disease 19 (COVID-19) (1). The rapid, ongoing evolution of SARS-CoV-2 has produced variants of concern, such as omicron, which can escape immune responses and evade antibody therapies (2–4). Therapeutic molecules (i.e., antibodies) that bind the spike protein will lose efficacy as new virions evolve under the selective pressure of these molecules. By contrast, as long as emerging variants continue to target the same receptor for cellular entry, a receptor-based therapy could provide broad, variant-independent protection. Such a valuable tool would provide immediate and long-term potential utility. The cell-surface receptor for SARS-CoV-2 is human angiotensin-converting enzyme 2 (ACE2) (5, 6), a receptor that has been exploited by other pathogenic coronaviruses including SARS-CoV-1 (7). ACE2 is a zinc carboxypeptidase (8, 9) that converts the 8-residue peptide angiotensin II (Ang II) to An-(1-7). Ang II is produced by angiotensin-converting enzyme (ACE), and the opposing activities of ACE and ACE2 regulate the renin-angiotensin system (RAS) (10). Delivery of an enzymatically inactive ACE2 as an anti-SARS-CoV-2 agent would potentially be less disruptive to this regulatory system. Soluble derivatives of ACE2 have been proposed for treating and preventing SARS-CoV-2 and are being explored in pre-clinical studies (11–18) and clinical settings (19). Notably, Shoemaker et al. have shown that ACE2 survives nebulization and that ACE2 delivered in an aqueous solution can protect mice from a laboratory-adapted strain of SARS-CoV-2 (13). The ideal formulation of such an ACE2 would enable rapid, large-scale purification of a protein that retains anti-viral efficacy following nebulization for delivery into the respiratory system.

**Fig 1:**
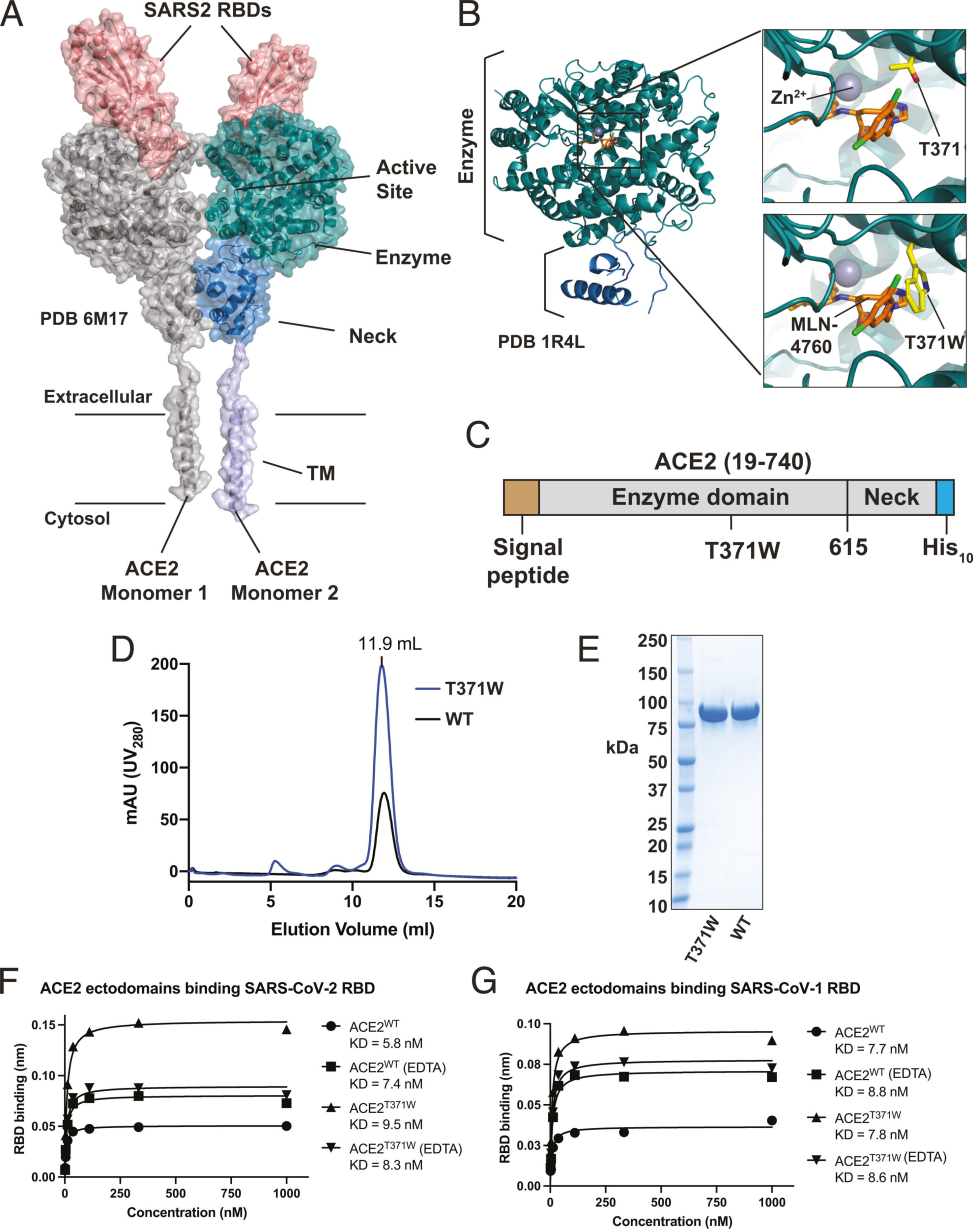
Design and characterization of a mutant dimeric ACE2. (**A**) Cryo-EM structure of full-length ACE2 bound to the RBDs of SARS-CoV-2 (PDB 6M17). One ACE2 monomer is depicted in gray. The other ACE2 monomer is depicted in purple, blue, and teal for the transmembrane (TM), Neck, and Enzyme domains, respectively. Bound RBDs are shown in red. Approximate positions of the membrane boundaries are indicated. (**B**) Structure-guided design of the active site T371W mutant. The crystal structure of the ACE2 ectodomain bound to MLN-4760 (PDB 1R4L) is shown on the left, with the domains colored as in panel **A**. Insets show active sites with MLN-4760, T371, and T371W mutations depicted with sticks. Zn^2+^ metal is shown with the gray sphere. (**C**) Schematic for the soluble ACE2 ectodomain construct for insect cell expression (**D**) Gel filtration traces for ACE2^WT^ and ACE2^T371W^ ectodomains grown in culture supplemented with 1 mM EDTA. NiNTA-purified protein samples were subjected to gel filtration over a superdex 200 column. (**E**) Coomassie-stained SDS-PAGE analysis of purified ACE2^T371W^ and ACE2^WT^ ectodomains. (**F**) Steady-state binding responses of the SARS-CoV-2 RBD to different versions of ACE2 from the BLI experiment. (**G**) Steady-state binding responses of the RBD of SARS-CoV-1 to different versions of ACE2 from BLI experiment.

Cryo-EM studies of full-length ACE2 show the protein is a dimer on the cell surface, where it binds the SARS-CoV-2 receptor binding domain (RBD) (Fig. 1A). The ectodomain of ACE2, corresponding to residues 19–740, can be subdivided into two domains, the Enzyme domain and the Neck domain. Most of the dimer contacts are between the two Neck domains of the ACE2 monomers, with additional interactions occurring between the ACE2 Enzyme domains. Each spike RBD engages with an ACE2 monomer away from the dimer interface. Similarly, the ACE2 active site is not part of the dimer interface or the RBD-binding surface (6) (Fig. 1A), and disrupting the ACE2 active site would not be expected to disrupt SARS-CoV-2 binding to ACE2.

Here, we developed a version of ACE2 with a space-filling mutation to the catalytic pocket that greatly reduces the protease activity of ACE2 and facilitates rapid purification of the secreted protein using one-step affinity chromatography. This protein retains the ability to bind the RBD of SARS-CoV-2 and SARS-CoV-1 and prevents viral infection *in vitro*. Aerosolized ACE2 delivered using a nebulizer apparatus prevented disease in K18-hACE2 mice. These data demonstrate proof-of-concept for the use of a modified, catalytically impaired ACE2 to prevent COVID-19 through aerosol delivery.

## RESULTS

To explore the use of nebulized ACE2 as a candidate for anti-COVID-19 therapy, we designed a version of soluble ACE2 that retained the virus-neutralizing properties of endogenous soluble ACE2 and was engineered to enable simple and rapid purification. Using the crystal structure of the ACE2 ectodomain bound to the inhibitor MLN-4760 as a guide (20), we designed a space-filling mutation within the active site, T371W, which is predicted to clash with MLN-4760 (Fig. 1B). This residue is not catalytic and does not participate in Zn^2+^ coordination. We reasoned that this mutation would sterically block substrate binding, which could prove advantageous for purification and reduce the enzymatic activity for the therapeutic candidate.

We generated baculovirus encoding a melittin signal peptide, the complete human ACE2 ectodomain (residues 19–740), and a C-terminal His_10_ tag (Fig. 1C). Comparing the expression of ACE2^WT^ and ACE2^T371W^ in Sf9 insect cells using anti-His and anti-ACE2 antibodies, we observed similar levels of secreted soluble ACE2 in the Sf9-conditioned supernatant (via anti-ACE2 antibody); however, the ACE2^WT^ culture had greatly decreased signal for the C-terminal His_10_ tag compared to ACE2^T371W^ (Fig. S1A). Because ACE2 is a zinc carboxy-peptidase, we reasoned that the soluble ACE2 ectodomains may proteolyze their own C-terminal His_10_ tag. Indeed, supplementing the Sf9 culture media with 1 mM EDTA rescued levels of the ACE2 His_10_ tag in the culture media (Fig. S1A). Previous structural biology studies on ACE2 with purification by nickel-nitrilotriacetic acid (NiNTA) resin used C-terminally tagged ACE2 consisting of the enzyme domain (residues 19–615) without the neck domain (residues 616–740) (21–26). The only structure containing the full soluble ectodomain utilized protein purified by a multi-step tag-less biochemical protocol (20). While the Neck domain was not fully modeled in that study due to poor electron density, symmetry expansion of PDB 1R4L shows the same dimeric interface between Necks characterized in the transmembrane ACE2 structure using cryo-EM (6). We speculate that the extended Neck domain may allow cleavage of the C-terminal His tag by ACE2, whether in *trans* or in *cis*. Regardless, preserving the ACE2 Neck domain is likely critical to preserve the dimeric form observed in full-length ACE2 (6).

We hypothesized that our T371W mutation might permit rapid and efficient large-scale purification of His-tagged, dimeric ACE2 ectodomain. To obtain comparable ACE2^WT^ and ACE2^T371W^ samples (avoiding C-terminal proteolysis), we supplemented the Sf9 culture with 1 mM EDTA and conducted His-tag purification of secreted protein using an EDTA-resistant IMAC resin. Both ACE2^WT^ and ACE2^T371W^ ectodomains could be purified in this manner, albeit with yields of ≤1 milligram per liter of culture. These proteins showed similar behavior on gel filtration chromatography (Fig. 1D and E). We characterized the enzymatic activity of these proteins using an established ACE2 fluorescent self-quenched peptide cleavage assay (27) where the substrate peptide Mca-APK(Dnp) is incubated with the enzyme and Mca fluorescence is monitored. Whereas ACE2^WT^ showed activity when supplemented with 10 µM ZnCl_2_, ACE2^T371W^ showed greatly reduced activity (Fig. S1B). Moreover, ACE2^WT^ was inhibited with 10 µM MLN-4760, but the residual activity of ACE2^T371W^ was not further inhibited by MLN-4760 (Fig. S1C). These measurements confirm that the T371W mutation disrupts the ACE2 active site. The residual activity observed with ACE2^T371W^ is consistent with the fact that this mutation is not disrupting a catalytic residue but was instead designed to project into the substrate-binding pocket and specifically predicted to clash with MLN-4760 (Fig. 1B), which we took as a proxy for a putative substrate.

The T371 residue is not part of the binding site for the RBD of SARS-CoV-1 or SARS-CoV-2. Nevertheless, we evaluated whether the T371W mutation or the expression of ACE2 in the presence of EDTA altered the ability of ACE2 to bind the RBDs of these viruses. Because of the very poor yields of ACE2^WT^ when expressed from normal media, we turned to BioLayer Interferometry (BLI), since we could biotinylate and capture small amounts of ACE2^WT^ expressed without EDTA in the culture media. We purified ACE2^WT^ and ACE2^T371W^, with each protein expressed in the presence or absence of 1 mM EDTA. Following affinity purification using NiNTA resin, proteins were biotinylated using NHS-ester chemistry and then subjected to gel filtration chromatography to remove unreacted biotin. We carried out BLI studies where immobilized biotin-ACE2 was dipped into wells containing the RBD of SARS-CoV-1 or SARS-CoV-2 in solution. The biosensors recorded dose-dependent binding, albeit with a somewhat weak signal likely resulting from the relatively small size of the RBDs compared to ACE2. The lowest binding responses were obtained using ACE2^WT^ expressed without EDTA, whereas the strongest binding responses were obtained using ACE2^T371W^ expressed without EDTA (Fig. 1F and G; Fig. S1D through G). Owing to the low signal with ACE2^WT^, confident kinetic fits could not be obtained. Steady-state analysis revealed all four ACE2 samples bound the RBDs of SARS-CoV-1 and SARS-CoV-2 with comparable affinities in the single-digit nanomolar range. These values are consistent with the K_D_ = 15 nM reported using surface plasmon resonance (SPR) measurements on SARS-CoV-2 and ACE2 ectodomains (28). The different responses were not due to unequal loading of ACE2 on the pins. Likewise, the addition of EDTA or ZnCl_2_ to the purified ACE2 did not alter the binding responses (not shown). Because the same RBD sample was used to measure binding to all forms of ACE2 in each experiment, we interpret the different response levels as reflecting changes to the availability of ACE2 binding sites resulting from the expression conditions and potentially the autocatalytic degradation of ACE2^WT^.

Next, we characterized the solution behavior of ACE2^T371W^. For all the remaining experiments, ACE2^T371W^ was expressed without supplementing the culture with EDTA, as this was no longer needed to protect the C-terminal His_10_ tag from proteolysis. In this format, we routinely obtained greatly enhanced yields of 10 milligrams per liter culture without further optimization. As expected, ACE2^T371W^ contains N-linked glycosylations (Fig. S2A). The purified protein is dimeric as judged by size exclusion chromatography-multi angle light scattering (SEC-MALS) analysis (predicted dimer MW = 172 kDa, experimental MW = 163 kDa) (Fig. S2B). The protein shows good thermal stability by differential scanning fluorimetry (DSF) with a T_m_ of ~48°C. ACE2^T371W^ is marginally less stable when the N-linked glycans are removed using PNGaseF (Fig. S2C and D ). We also measured the denaturation of the protein using circular dichroism spectroscopy (CD), which showed a single transition at ~50°C. Interestingly, the protein did not lose all secondary structure, even with temperatures up to 95°C (Fig. S2E).

**Fig 2:**
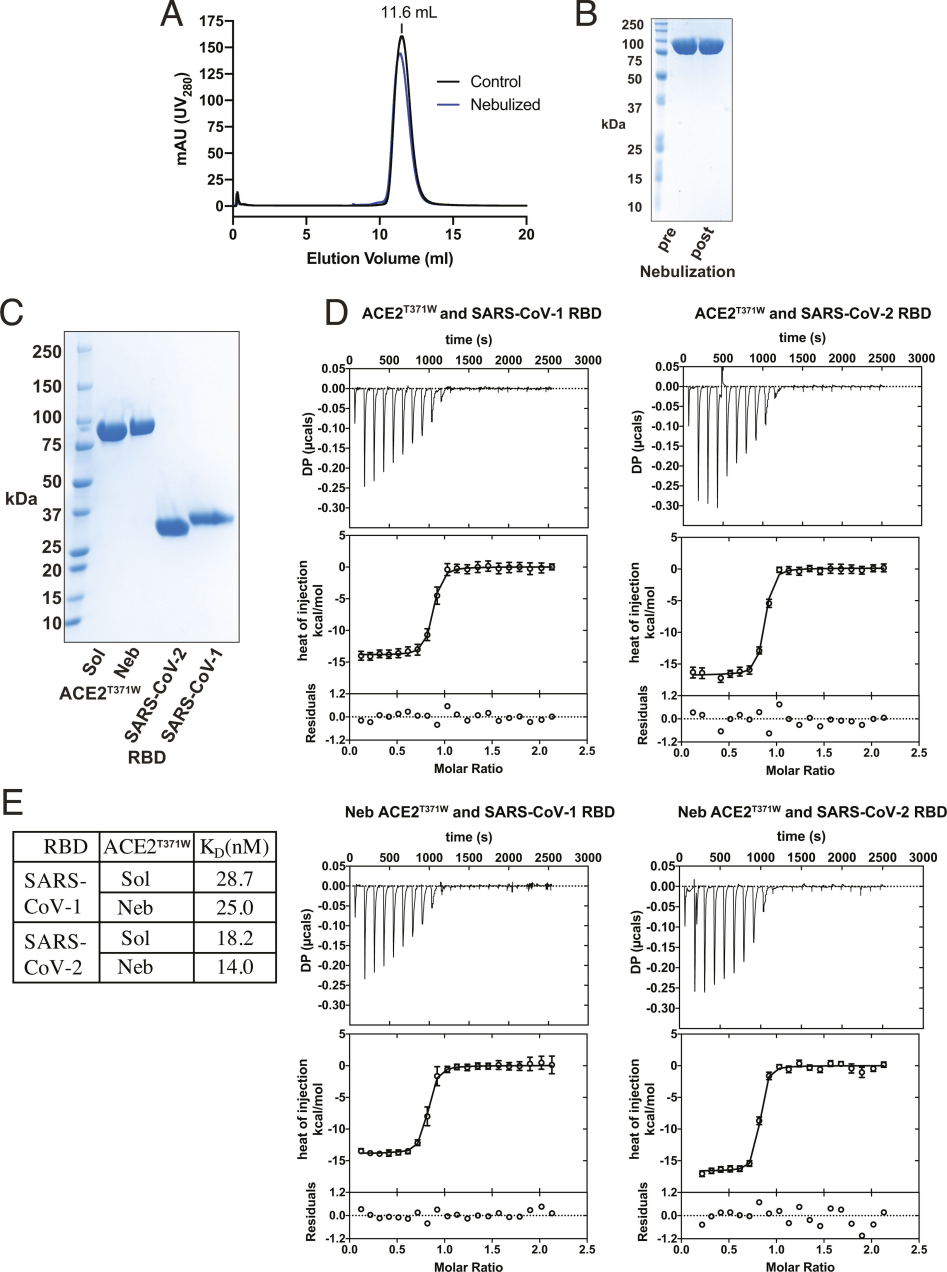
Nebulized ACE2^T371W^ retains binding to the RBDs of SARS-CoV-1 and SARS-CoV-2. (**A**) Gel filtration traces for ACE2^T371W^ ectodomain samples treated with 1% wt/vol PEG-8000 were collected before nebulization (Control) or after nebulization (Nebulized). (**B**) Coomassie-stained SDS-PAGE analysis of the samples from panel **A**. (**C**) Coomassie-stained SDS-PAGE analysis of protein samples for ITC experiments. (**D**) Isotherms for ACE2^T371W^ and RBD isothermal titration calorimetry experiments. The upper panel shows the SVD-corrected thermograms from NITPIC; the middle panel displays the integrated data points with their respective estimated error bars, and the solid line results from the fit. The lower panel depicts the residuals between the data and the fit lines. (**E**) Affinity values derived from ITC data. The binding reactions between sACE2 and the RBD of either SARS-CoV-2 or SARS-CoV-1 were measured from four or two independent purifications, respectively. The binding reactions between nebACE2 and the RBD of either SARS-CoV-1 or SARS-CoV-2 RBDs were measured from one purification.

Next, we tested whether the dimeric ACE2^T371W^ survives nebulization. ACE2^T371W^ collected before or after nebulization with 1% wt/vol PEG-8000 as excipient (hereafter soluble [sACE2^T371W^] and nebulized [nebACE2^T371W^], respectively) displayed identical gel filtration profiles, and nebACE2^T371W^ remained intact as shown by Coomassie-stained SDS-PAGE (Fig. 2A and B). To test whether nebulized ACE2^T371W^ retains binding to the RBDs of SARS-CoV-1 and SARS-CoV-2 spike proteins, we employed isothermal titration calorimetry (ITC). The RBDs of these viruses were expressed as secreted FLAG-tagged proteins from Sf9 insect cells and purified by anti-FLAG chromatography and gel filtration (Fig. 2C). ITC experiments were conducted to measure the heat of binding between ACE2^T371W^ and the RBDs (Fig. 2D). Strong binding isotherms were observed for binding to both SARS-CoV-1 and SARS-CoV-2 RBDs, and the affinity constants for sACE2^T371W^ and nebACE2^T371W^ were nearly identical (Fig. 2E) and consistent with the BLI experiments described above (Fig. 1F through G).

**Fig 3:**
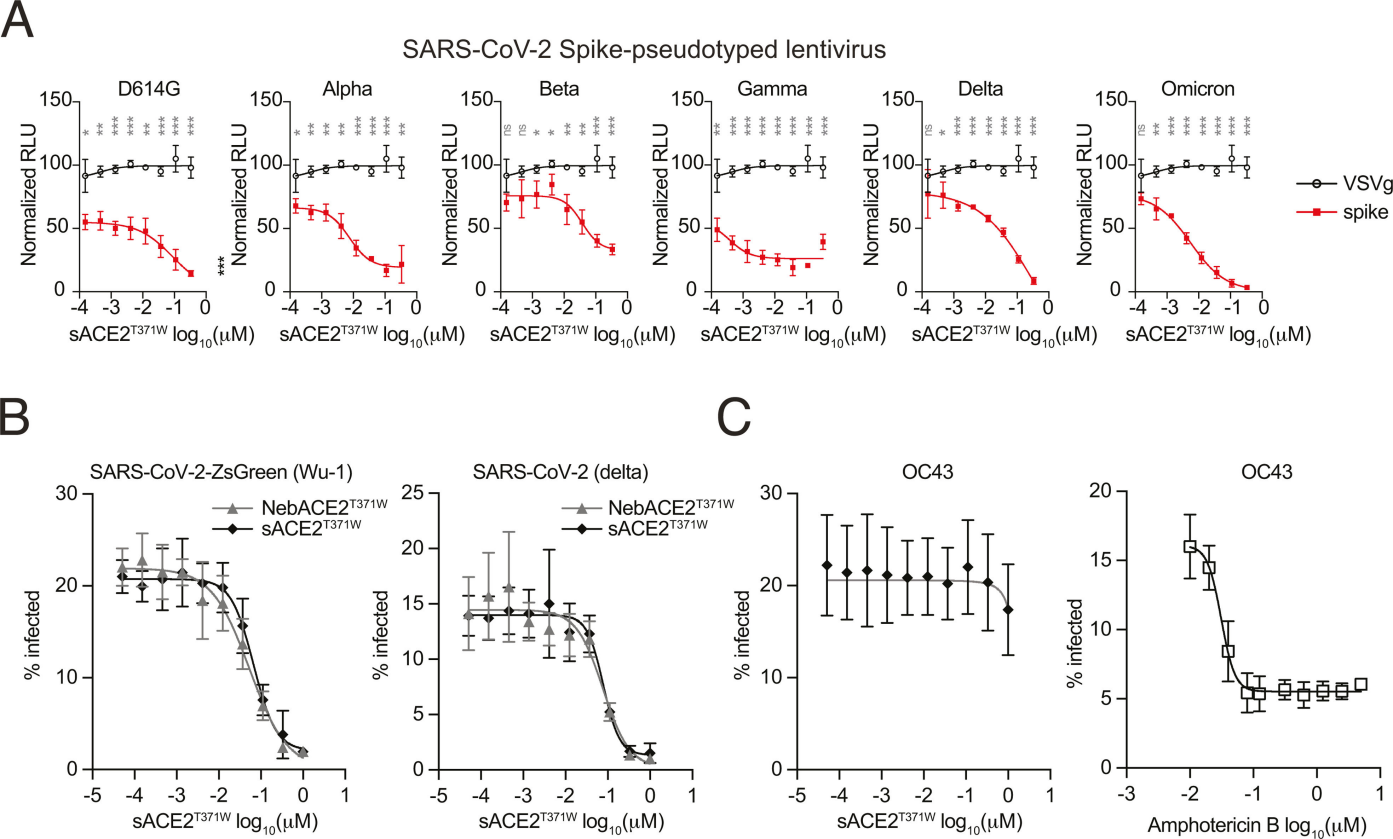
sACE2^T371W^ and nebACE2^T371W^ inhibit spike-pseudotyped lentivirus and SARS-CoV-2. (**A**) Infectivity of luciferase-expressing SARS-CoV-2 spike-pseudotyped lentiviruses (red) or VSV-G-pseudotyped lentivirus (black) in the presence of varying concentrations of sACE2^T371W^. An equal volume of sACE2^T371W^ and spike-pseudotyped or VSVg-pseudotyped lentivirus were incubated for 30 minutes at 37°C, followed by the addition of 10,000 Huh7.5mutACE2 cells. 48 hours later, cells were lysed and luciferase activity was detected by the Luciferase Assay System. *N* = 3 biological replicates. Statistical significance was determined by an unpaired *t*-test. *P < 0.05; **P < 0.01; ***P < 0.001. (**B**) Infectivity of SARS-CoV-2-Wu-1-zsGreen or delta variant in the presence of varying concentrations of sACE2^T371W^ (black) or nebACE2^T371W^ (gray). sACE2^T371W^ or nebACE2^T371W^ was incubated with 1–2 MOI of SARS-CoV-2 variants for 30 minutes at 37°C. Each ACE2/virus complex was transferred to 100,000 A549-TMPRSS2-ACE2 cells, followed by incubation at 37°C. At 7 hours post-infection, cells were fixed with 4% PFA. Delta variant samples were stained with SARS-CoV/SARS-CoV-2 Nucleocapsid antibody. Viral infectivity was quantified by flow cytometry. *N* = 3 biological replicates. (**C**) Infectivity of human coronavirus OC43 in the presence of varying concentrations of sACE2^T371W^ or amphotericin B. sACE2^T371W^ (right) or amphotericin B (left) was incubated with 1 MOI of human coronavirus OC43 for 30 minutes at 33°C. Each ACE2 or amphotericin/virus complex was transferred to 80,000 A549-TMPRSS2-ACE2 cells, followed by incubation at 33°C. At 24 hours post-infection, cells were fixed with 1% PFA. Samples were stained with anti-coronavirus group antigen antibody, nucleoprotein of OC-43, clone 542–7D. Viral infectivity was quantified by flow cytometry. *N* = 3 biological replicates.

We next tested whether ACE2^T371W^ inhibits viral infection, starting with a tractable assay that could be performed in Biosafety Level 2 conditions. We used replication-defective HIV-1-based lentivirus particles pseudotyped with the spike protein from several SARS-CoV-2 variants (D614G, alpha, beta, gamma, delta, omicron), or with the glycoprotein from vesicular stomatitis virus (VSV-G) as a control. The lentiviruses encode firefly luciferase as a readout of viral transduction. Huh7.5 cells ectopically expressing ACE2 were transduced with lentiviruses in the presence of sACE2^T371W^. 48 hours later, luciferase signals from cell lysates were quantified, and data were normalized to the levels of luciferase generated from untreated, transduced cells. In the presence of sACE2^T371W^ concentrations ranging from 333nM to 0.15 nM, VSV-G-pseudotyped lentivirus was not inhibited (Fig. 3A). By contrast, spike-pseudotyped lentiviruses incubated with sACE2^T371W^ were inhibited in a dose-dependent manner. Inhibitory effects varied across the spike variants, with suppression ranging from 65% to 95% at the maximum dose of 333 nM sACE2^T371W^.

We next tested the inhibitory potential of ACE2^T371W^ against authentic SARS-CoV-2 in A549 cells expressing ACE2 and TMPRSS2 in a Biosafety Level 3 setting. Over a 10-point dilution series starting at 1 µM, we found that sACE2^T371W^ strongly inhibited a ZsGreen-expressing recombinant SARS-CoV-2 (Wu-1 strain) and a clinical isolate of the delta variant (Fig. 3B). The human coronavirus OC43, which uses sialic acid instead of ACE2 for entry (29), was not inhibited by sACE2^T371W^ (Fig. 3C). As a control for OC43 inhibition, we used amphotericin B and observed dose-dependent inhibition, as previously reported (Fig. 3C) (30). Notably, SARS-CoV-2 was also inhibited by the nebulized form of ACE2^T371W^ (Fig. 3B). The inhibitory potential of ACE2T^371W^ was similar to that of ACE2^WT^ when tested against the Wu-1 strain (Fig. S3A). In these cell culture studies, treatment of Huh7.5-mutACE2 and A549-TMPRSS2-ACE2 with ACE2^WT^ or ACE2^T371W^ had little to no effect on cell viability or cell toxicity, as measured by ATP assay and lactate dehydrogenase (LDH) release, respectively (Fig. S3B and C). This prompted us to examine whether aerosolized ACE2^T371W^ could be administered to mice via the respiratory route to suppress SARS-CoV-2 infection *in vivo*.

**Fig 4:**
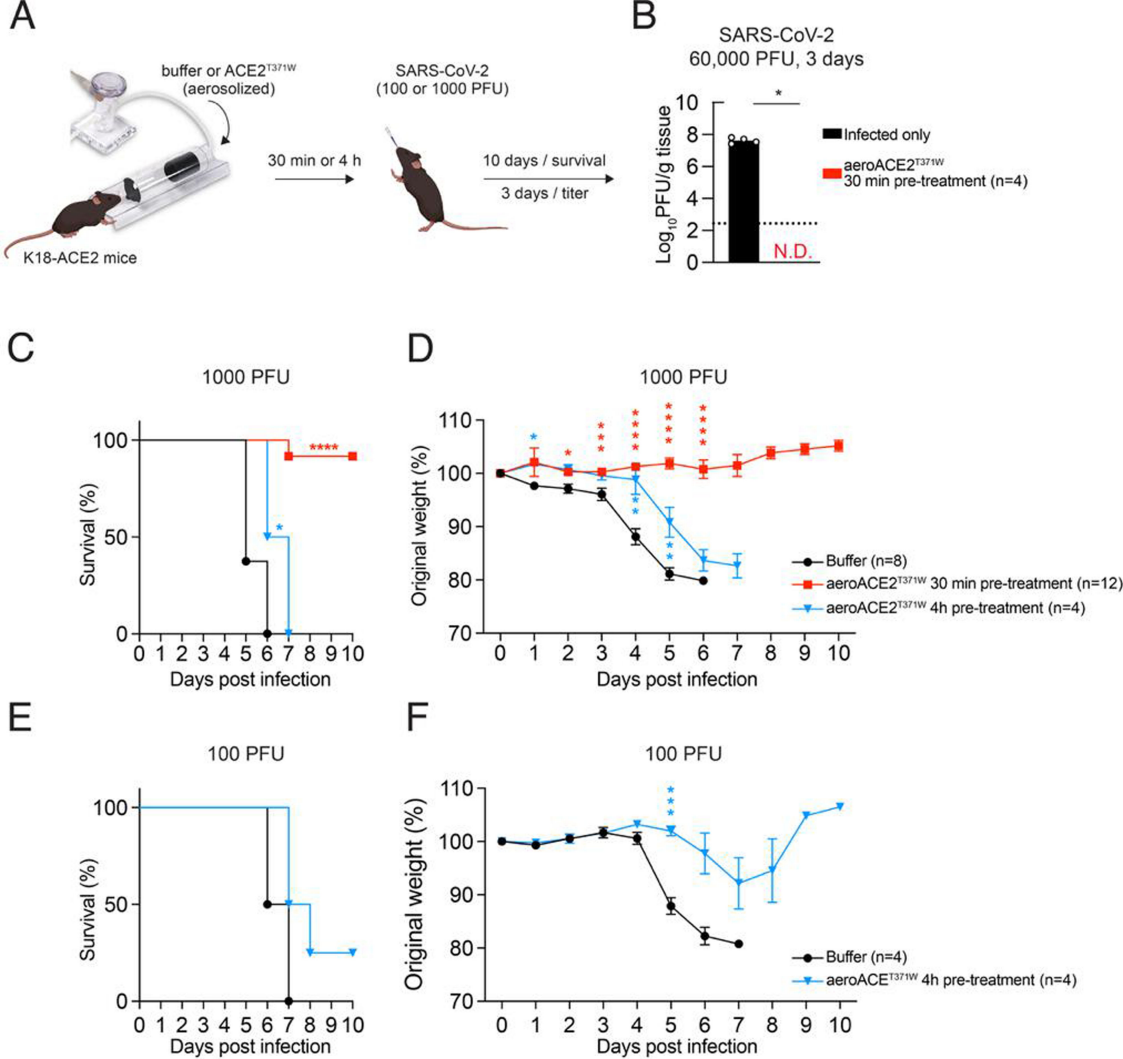
sACE2^T371W^ inhibits SARS-CoV2 replication and increases survival rates in mice. (**A**) Experimental scheme to demonstrate aerosolized treatment and intranasal (IN) infection of K18-hACE2 mice. (**B**) K18-hACE2 mice were treated intranasally with sACE2^T371W^ 30 minutes prior to infection or infected alone with 60,000 PFU SARS-CoV-2 P.1 and assessed for viral burden in the lungs by plaque assay at 3 days post-infection. All male mice were used for this experiment. *n* = 4 mice per group. (**C** and D) Survival and daily weight of K18-hACE2 mice nebulized with sACE2^T371W^ either 30 minutes prior to or 4 hours prior to Intranasal (IN) infection with 1,000 PFU P.1 SARS-CoV-2. Data represent means from *n* = 8 buffer-treated mice (2M/6F), *n* = 12, 30 minutes prior to sACE2^T371W^ treatment (6M/6F), and *n* = 4, 4 hours prior sACE2 treatment (4F) (**E** and F) Survival and daily weight of K18-hACE2 mice nebulized with sACE2^T371W^ 4 hours prior to Intranasal (IN) infection with 100 CFU P.1 SARS-CoV-2. Data represent means of *n* = 4 for each group and all female mice were used. Statistical significance was determined by Mann-Whitney test (B), log-rank (Mantel-Cox) tests (C) and unpaired *t*-tests (D and F) *P < 0.05, **P < 0.01, ***P < 0.001, ****P < 0.0001.

For *in vivo* studies, we used the K18-hACE2 mouse model, in which human ACE2 is expressed under the control of the keratin 18 promoter (31). These mice are exquisitely sensitive to SARS-CoV-2 (32, 33). The experimental approach included a nebulizer delivery apparatus coupled to an animal holder with an integrated nose cone (Fig. 4A). This allows for the delivery of aerosolized material toward the animal’s respiratory zone. We refer to this form of ACE2^T371W^ as “aeroACE2^T371W^” to distinguish it from the nebACE2^T371W^ generated for cell culture experiments. Mice were treated with buffer alone or aeroACE2^T371W^ 30 minutes or 4 hours prior to infection with 1,000 plaque forming units (PFU) SARS-CoV-2 gamma variant (P.1), which causes severe disease in K18-ACE2 mice (34). Weight was monitored daily, and mice were euthanized when they reached 80% of the starting body weight. In the buffer-treated group, all mice lost weight and were euthanized on day 5 or 6 (Fig. 4B through D). Remarkably, in the cohort treated with aeroACE2^T371W^ 30 minutes prior to infection, nearly all mice survived, with only one death on day 7. In a separate cohort, we quantified viral titers in lungs from mice infected for 3 days under the same treatment conditions. Buffer-treated mice had over 7log_10_ PFU per gram tissue, whereas aeroACE2^T371W^ -treated mice did not have detectable virus in their lungs at this time point (Fig. 4B). We next pretreated mice with aeroACE2^T371W^ for 4 hours prior to infection to determine if the prophylactic effect would be temporally extended. All mice succumbed to infection, with only a slight delay of 1–2 days in their demise relative to the control group (Fig. 4C and D). These data suggest that prophylaxis with aeroACE2^T371W^ relatively soon before SARS-CoV-2 exposure provides strong protection from infection and disease.

As the mice treated with aeroACE2^T371W^ 4 hours prior to infection still succumbed, we asked whether a lower viral dose could reveal a protective effect for aeroACE2^T371W^ at this time point. We repeated the experiment at 100 PFU SARS-CoV-2 and found that 100% of control mice and 75% of aeroACE2^T371W^-treated mice succumbed to infection (Fig. 4E). While the survival difference was not statistically different given the small cohort, weight loss between the groups was statistically significant (Fig. 4F). We extended the prophylactic treatment further by treating mice with aeroACE2^T371W^ 1 day, 2 days, or 1 and 2 days prior to infection. We observed no difference in survival or weight loss relative to untreated mice (Fig. S3D and E). We also treated mice “therapeutically” by administering aeroACE2^T371W^ 30 minutes after infection. Compared to untreated mice, the treated cohort lost less weight and had a considerable delay in their survival curve (Fig. S3D and E). However, these mice died by day 10.

## DISCUSSION

In this work, we demonstrate three advances toward using ACE2 as an aerosolized therapeutic for COVID-19. First, we developed an ACE2 variant that enables rapid, efficient purification of the full ACE2 ectodomain without C-terminal proteolysis. Second, we show this protein can be nebulized and recovered intact with no loss of RBD binding and prevents infection by multiple SARS-CoV-2 variants *in vitro*. Finally, we show that aerosolized ACE2^T317W^ prevents disease in an animal model of COVID-19.

The full ACE2 ectodomain has been purified as a secreted protein without an affinity tag using several biochemical separation methods (27). This protein crystallizes via dimeric interactions between symmetry-related monomers that resemble the dimerization seen in the full-length ACE2 dimer (6, 20). By contrast, secreted constructs containing the Enzyme domain but without the Neck domain have been purified for structural studies using standard NiNTA affinity with a C-terminal His tag (21–26). We found the full ACE2^WT^ ectodomain proteolyzes its own C-terminal His_10_ tag, and this His_10_ tag could only be used for purification when the protein was expressed in cells treated with 1 mM EDTA to inhibit metalloprotease activity (Fig. S1A). We have not determined the extent to which ACE2^WT^ proteolyzes C-terminal portions of the Neck domain in addition to the His tag, but we were eager to preserve this region as it is an important mediator of ACE2 ectodomain dimerization. A soluble dimeric ACE2 construct more faithfully mimics the cell surface receptor, and the avidity effect conferred by dimerization should enhance the ability of soluble ACE2 to compete for virus binding. Proteolytic stability of the C-terminal His_10_ tag ensures the purification of soluble ACE2 with the dimerizing Neck domain intact and allows a single-step purification from culture media.

The T371W mutation was designed to project into the substrate binding pocket of ACE2 and prevent substrate binding (Fig. 1B). The introduced Trp residue is predicted to occupy the same area as the inhibitor MLN-4760 molecule in the enzyme’s active site (20). The T371W mutation does not change the solution behavior of the protein compared to ACE2^WT^ (Fig. 1D), and the mutant ACE2 has the expected dimer MW in solution (Fig. S2B). The T371W mutation largely abolishes the enzymatic activity of ACE2 (Fig. S1A through C), and the residual activity is not inhibited by MLN-4760, confirming the disruption of the substrate binding pocket by this modification (Fig. S1C). We confirmed this mutation does not alter the ability of ACE2 to bind the RBD of SARS-CoV-1 or SARS-CoV-2 (Fig. 1F and G) and it inhibits the infection of cells by the Wu-1 strain of SARS-CoV-2 (Fig. S3A). ACE2^T371W^ could be nebulized with >90% recovery and no change in measurable biophysical behavior. The nebulized protein bound the RBDs of SARS-CoV-1 and SARS-CoV-2 equivalently to non-nebulized ACE2^T371W^, with similar affinities to those reported for the WT ACE2 interaction here (Fig. 1F and G; 2D and E) and through SPR studies (28). Nebulized or not, our mutant soluble ACE2 inhibited infection by viruses containing an array of spike variants (Fig. 3).

The aerosolized ACE2^T371W^ prevented disease when given prophylactically 30 minutes before infection (Fig. 4). However, the protein was not as effective when administered at earlier times before viral infection (Fig. S3). We have not measured the rate of clearance of the nebulized ACE2^T371W^ *in vivo*. It is possible that formulations that reduce the rate of clearance of the nebulized ACE2 in the respiratory system would improve its efficacy for longer times prior to exposure. The protein was also not effective when given as a treatment post-infection (Fig. S3). While we were preparing this paper, Onodera et. al. reported that the administration of nebulized, recombinant ACE2 post-infection with SARS-CoV-2 could attenuate viral load and lung injury in male mice (18). This efficacy may be due to the effect of the WT ACE2 on the RAS system and further exploration is warranted to delineate the effects of ACE2 as a viral decoy or as an enzyme. Further *in vivo* studies will be required to determine whether this protein has higher prophylactic or treatment efficacy in an animal model expressing lower unmodified levels of endogenous ACE2. Further studies can also address whether the residual activity of our ACE2 protein alters the RAS pathway in animal lungs. In these initial experiments, the protein appeared well tolerated by the animals. Our study is additionally limited as we did not address lung pathology or the fate of viral particles in sACE2-treated mice. In mice pre-treated with sACE2 for 30 minutes, we observe nearly complete protection from lethality and little to no weight loss. However, it is possible that the lungs of these mice still exhibit overt pathology. In addition, although no virus was detected in the lungs of mice pre-treated for 30 minutes with sACE2, we did not examine the fate of the viral inoculum. It is possible that the virus is being taken up by phagocytic cells and activating immune pathways. Additional histopathological and immune activation studies would be needed to address these limitations. Our work demonstrates the utility of an aerosol delivery system to deliver a therapeutic protein and prevent infection in K18-ACE2 mice by SARS-CoV-2. The modified ACE2 described herein is a potential anti-viral agent for any virus that exploits human ACE2 for cellular entry.

## MATERIALS AND METHODS

See Table 1 for reagents and tools.

**TABLE 1:** Reagents and tools

Reagent or resource	Reference or source	Identifier or catalog number
Experimental models		
K18-hACE2 (*M. musculus*)	Jackson Lab	B6.Cg-Tg(K18-ACE2)2Prlmn/J Stock # 034860
Huh7.5_mutACE2 (*H. sapiens*)	This study. Huh7.5 parental from Rice Lab.	
A549-TMPRSS2-ACE2	(35)	
Sf9 cells (*S. frugiperda*)	Expression Systems Inc.	94–001S
Vero-E6-C1008 (*C. aethiops*)	ATCC	CRL-1586
HEK-293T (*H. sapiens*)	Rice Lab	
Recombinant DNA		
pFastBac-ACE2	This study	
pSCRBBL-Fluc	(36)	
pLV-Spike (and variants)	InvivoGen	
pCMV.gag-pol	Rice Lab	
pCMV.VSVg	Rice Lab	
Antibodies		
Anti-ACE2	Abcam	Cat# ab108252
Anti-His His.H8	Millipore Sigma	Cat# 05–949
Anti-coronavirus group antigen antibody, nucleoprotein of OC-43, clone 542–7D	Millipore Sigma	Cat# MAB9013
SARS-CoV/SARS-CoV-2 nucleocapsid antibody	Sino Biological	RRID Number: AB_2827974
Chemicals, enzymes, and other reagents		
MLN-4760	MedChemExpress	Cat # HY-19414
Mca-APK(Dnp)-OH trifluoroacetate	Sigma-Aldrich	Cat # SML2948
Ni-penta agarose	Fisher Scientific	Cat # NC1210120
Amphotericin B	Thermo Fisher	Cat # 15290–026
EZ-link NHS-biotin	Thermo Fisher	Cat # 20217
Software		
NITPIC	(37)	
SEDPHAT	(38)	
GUSSI	(39, 40)	
GraphPad Prism 10	www.graphpad.com	
FlowJo	www.flowjo.com	
Other		
Luciferase Assay System	Promega	Cat # E1501
CellTiter-Glo	Promega	G7571
LDH-Glo	Promega	J2380
BD Cytofix/Cytoperm Kit	BD Biosciences	RRID: AB_2869008
Flow cytometer	Stratedigm	S1000EON
Luminometer	Berthold Technologies	Centro XS^3^ LB 960 Microplate Luminometer

### Cells

HEK-293T cells were maintained in DMEM supplemented with 10% FBS and 1× non-essential amino acids. A549-TMPRSS2-ACE2 cells (a gift from C. Rice (35)) were maintained in DMEM supplemented with 10% FBS, 1× non-essential amino acids, and 0.4 mg/mL Geneticin. Huh7.5 cells were transduced with a lentiviral vector (SCRBBL) expressing a catalytically inactive human ACE2, selected with 10 µg/mL Blasticidin, and maintained in DMEM supplemented with 10% FBS and 1× non-essential amino acids. Spodoptera frugiperda (Sf9) cells were grown in ESF 921 media while shaking at 140 rpm at 27°C. The cell line was not further authenticated and was not tested for mycoplasma contamination.

### Expression constructs and baculovirus production for protein purification

The pFastBac-ACE2 plasmid was created using Gibson assembly methods to insert a synthetic dsDNA block into the pFastBac-1 plasmid that was linearized with BamHI and XhoI. The resulting construct encodes a Melittin signal peptide, the full human ACE2 ectodomain (residues 19–740), a TEV recognition site (ENLYFQG), a TG spacer, and 10 His residues. Mutations were introduced through standard PCR mutagenesis methods. Mutagenesis primer sequences are available upon request.

Baculovirus was produced in Sf9 cells using the Bac-to-Bac Baculovirus Expression System (ThermoFisher) according to the manufacturer’s instructions.

### Expression and purification of ACE2 ectodomains

Sf9 cells at a density of 2 million cells/mL were infected with 20 mL of P2 baculovirus per liter of culture and then cultured for 96 hours. Cells were pelleted by centrifugation for 10 minutes at 6,000 × *g* at 4°C and the supernatants were either used directly or else supplemented with 20% glycerol and frozen at −80°C until purification. To purify the ACE2 ectodomains, the supernatants were supplemented with protease inhibitors (160 µg/mL benzamidine, 100 µg/mL leupeptin, 1 mM PMSF, and 1 μM E-64), and further clarified by filtration through a 0.2-μm filter. His-tagged ACE2 ectodomains were purified by immobilized metal ion affinity chromatography using Ni-penta agarose resin. Supernatants were passed over the resin using gravity. The resin was washed in 50 mM HEPES (pH 7.5), 150 mM NaCl, and 20 mM imidazole (pH 7.5). The ACE2 ectodomains were eluted in the same wash buffer supplemented with imidazole to a final concentration of 300 mM. For animal experiments, the protein was subjected to gel filtration in a Superdex 200 column (GE) equilibrated in sterile phosphate buffered saline. Eluted samples were concentrated to ~10 mg/mL and then supplemented with PEG-8000 to a final concentration of 1% wt/vol. For experiments other than the peptide cleavage assay, the protein was purified over the same column equilibrated in 20 mM HEPES (pH 7.5) and 150 mM NaCl. For the peptide cleavage assay, ACE2 proteins were purified as described above except that 1 mM EDTA was added to the Sf9 cultures at the time of infection and the final gel filtration buffer was 150 mM NaCl, 50 mM HEPES (pH 7.5), and 100 µM EDTA.

### BioLayer interferometry

ACE2 proteins purified by NiNTA chromatography were biotinylated using EZ-link NHS-biotin. Excess biotin was removed by subjecting the proteins to gel filtration as described above. An Octet instrument (Sartorius) was used to measure protein-protein interactions through BioLayer Interferometry. All reactions were carried out in an assay buffer consisting of PBS supplemented with 0.1% BSA and 0.005% Tween-20. Biotinylated ACE2 proteins were immobilized using streptavidin-coated (SA) biosensors, washed, and then dipped into wells with the RBD from either SARS-CoV-1 or SARS-CoV-2 at concentrations ranging from 1,000 nM to 1.4 nM. The association step lasted 180 seconds, after which pins were dipped into a buffer well and dissociation was measured for 180 seconds. Data were processing used double-reference subtraction using the signal from immobilized protein dipping into the buffer (no RBD) and an unloaded pin dipping into each concentration of RBD. Owing to the relatively weak binding signal and potential complication from the dimeric ACE2, kinetic analysis is not reported. Affinities were derived from steady-state binding responses using the Octet Data Analysis software.

### SEC-MALS

A Sephadex 200 Increase 10/300 size-exclusion column was equilibrated with a gel filtration buffer. 400 µg of the sample at 2 mg/mL was injected and chromatographed at a flow rate of 0.5 mL/min. Data were recorded on a Shimadzu UV detector, a Wyatt TREOS II light-scattering detector, and a Wyatt Optilab t-REX differential refractive-index detector, which were all calibrated with a BSA standard. SEC-MALS data were analyzed with ASTRA version 7.3.0.11.

### Circular dichroism spectroscopy

Circular dichroism spectroscopy was performed on MBR’s Jasco J-815 CD Spectrometer. Samples were prepared and placed in a 0.1 cm CD cuvette. As a first step, spectra were collected on the samples from 190 to 250 nm. 208 nm and 222 nm were selected as wavelengths to monitor the CD as a function of temperature, which was varied from 25°C to 95°C at a ramp rate of 2°C/min. Only the data taken at 222 nm were analyzed.

### Isothermal titration calorimetry

ITC experiments were carried out in a Malvern iTC200 calorimeter at 20°C. A first injection volume of 0.5 µL was employed, followed by 20 injections of 1.9 µL each; all injection rates were 0.5 µL/s. The stir rate was 750 rpm, and the reference power was 5.0 μcal/s. The time between injections was 120 s, with a data-filtering period of 5 s. ACE2 was included in the cell at a concentration of 11.8 µM and RBD was included in the syringe at a concentration of 120 µM. The data were integrated, analyzed, and illustrated using NITPIC (37), SEDPHAT (38), and GUSSI (39, 40), respectively.

### Thermal stability analysis

Differential scanning fluorimetry (DSF) experiments were conducted using a BioRad CFX96 Real-Time System machine. 20 µL reactions were set up with a protein concentration of 5 µM in gel filtration buffer that was supplemented with SYPRO Orange dye at a final concentration of 5×. The temperature was increased from 20°C to 95°C at a rate of ~1°C per minute and fluorescence was recorded in all channels. The dye fluoresced most strongly in the HEX channel, and the data were used for all analyses.

### ACE2 peptide cleavage assay

The fluorescent peptide cleavage assay was set up with final concentrations of 0.15 nM protein, 150 mM NaCl, 50 mM HEPES, 0.01% Nonidet P-40, and 1 µM EDTA. Where indicated, ZnCl_2_ and MLN-4760 were added to a final concentration of 10 µM. The protein was incubated in these conditions at RT for 30 minutes and then the reaction was initiated by the addition of Mca-APK(Dnp)-OH trifluoroacetate to a final concentration of 50 µM. The reaction was analyzed at RT at the indicated time points using a CLARIOstar plate reader (BMG LABTECH) with excitation at 320 nm and emission at 405 nm.

### Immunoblot analysis

Samples were mixed with 5× SDS-PAGE loading buffer, heated at 37°C for 5 minutes, and subjected to 10% SDS-PAGE. Proteins were transferred to nitrocellulose filters using the Bio-Rad *Trans* Blot Turbo system and immunoblotted with a 1:1,000 dilution of either monoclonal anti-His clone His.H8 or anti-ACE2 (Abcam ab108252). Bound antibodies were visualized by chemiluminescence (SuperSignal West Pico Chemiluminescent Substrate, Thermo Scientific, Waltham, MA) using a 1:5,000 dilution of anti-mouse IgG or anti-rabbit IgG conjugated to horseradish peroxidase (Jackson ImmunoResearch Laboratories, Inc., West Grove, PA). The filters were exposed to Blue X-ray Film (Phoenix Research Products, Pleasanton, CA).

### Viruses

The virus from infectious clone pCC1-4K-SARS-CoV-2-Wuhan-Hu-1-ZsGreen (a gift from S. Wilson) was generated and propagated as previously described (41). SARS-CoV-2, isolate hCoV-19/USA/MD-HP05647/2021 (Lineage B.1.617.2; Delta variant) (BEI), was propagated as previously described for other SARS-CoV-2 variants (41). SARS-CoV-2 (hCoV-19/Japan/TY7-503/2021, P.1) was obtained from BEI Resources and inoculated onto Vero-E6-C1008 (ATCC, VA) plated at a density of 7 × 10^6^ cells in a 175 cm^2^ tissue culture flask in 2% FBS (Gibco, MA)/1× non-essential amino acids (Gibco, MA)/MEM (Gibco, MA) for 45 minutes at 37°C. The viral inoculum was removed and replaced with 2% FBS (Gibco, MA)/1× non-essential amino acids (Gibco, MA)/MEM (Gibco, MA) and cells were incubated for 3 days at 37°C before supernatants were clarified by centrifugation at 1,000 × *g* for 5 minutes before aliquoting and storage at −80°C. Viral titer was determined by plaque assay on Vero-E6-C1008 cells (ATCC, VA). In brief, cells were plated at 6.5 × 10^5^ cells per well of a six-well plate and infected with six dilutions from a 10-fold dilution series in 1% FBS (Gibco, MA)/1× non-essential amino acids (Gibco, MA)/MEM (Gibco, MA) for 30 minutes at 37°C. Inoculum was then removed and replaced with Avicel overlay medium (5% FBS [Gibco, MA]/1× penicillin-streptomycin [Gibco, MA]/1× GlutaMAX [Gibco, MA]/1× modified Eagle medium, Temin’s Modification #11935-046 [Gibco, MA)]/1.2% Avicel RC-591 [DuPont, DE]). Cells were incubated for 3 days at 37°C before the overlay medium was removed and cells were fixed with 4% PFA (Sigma-Aldrich, MA) for 30 minutes at room temperature. Fixative was then removed, and cells were stained for at least 1 hour with crystal violet (0.2% crystal violet [Sigma-Aldrich, MA]/20% ethanol [Sigma-Aldrich, MA]) before removal and plaque enumeration. Human coronavirus OC43 (ATCC strain VR-1558) was propagated as previously described (42). Lentivirus and OC43 infections were performed in a Biosafety Level 2 (BSL2) facility and SARS-CoV-2 infections were performed in a Biosafety Level 3 (BSL3) facility, each equipped with the appropriate safety features to prevent bio-hazardous exposure or unintentional pathogen release. Experiments and biosafety protocols were reviewed and approved by the Institutional Biosafety Committee, according to guidelines provided by the UT Southwestern Office of Business and Safety Continuity.

### Lentiviral pseudoparticle production

Spike-pseudotyped HIV-1-based lentiviral vectors were generated by co-transfecting 293T cells with expression plasmids pSCRBBL-Fluc (36), pLV-spike (D614G variant, Alpha B.1.1.7 variant, Beta B.1.351 variant, Gamma P.1 variant, Delta B.1.617.2 variant, Omicron BA.4 & BA.5 variant) (Invivogen), and pCMV.gag-pol using XtremeGene9. Six hours post-transfection, media was replaced with DMEM containing 3% FBS and 1× non-essential amino acids. Supernatants were collected at 48 and 72 hours, pooled, supplemented with 20 mM HEPES and 4 ug/mL Polybrene, and aliquoted. Control lentiviruses were generated the same way but pseudotyped with VSV glycoprotein using plasmid pCMV.VSVg.

### Ten dilution drug curves and transduction with SARS-CoV-2 pseudotyped lentivirus

A threefold dilution series of sACE2^T371W^ starting at 333 µM was diluted in media containing DMEM, 3% FBS, 1× NEAA, 20 mM HEPES, and 4 µg/mL Polybrene. An equal volume of each sACE2^T371W^ dilution was incubated with an equal volume of each spike-pseudotyped lentivirus or VSVg-pseudoyped control for 30 minutes at 37°C. 10,000 Huh7.5mutACE2 cells were added to each well and incubated for 48 hours. Media were aspirated and 20 μL of 1× passive lysis buffer was added to each well, mixed on an orbital shaker at 900 rpm for 1 minute, and stored at −80°C. Luciferase activity was detected by the Luciferase Assay System and quantified on a Berthold Centro XS^3^ LB 960 luminometer.

### ACE2 inhibitory studies in cell culture

A549-TMPRSS2-ACE2 cells were plated at a density of 80,000–100,000 c/well. A threefold dilution series of sACE2^WT^, sACE2^T371W^, or nebACE2^T371W^, starting at 1 µM, was diluted in infection media containing 1% FBS and 1× NEAA. A twofold dilution series of Amphotericin B, starting at 5 µM was used as a positive inhibition control in hOC43 assays. An equal volume of each dilution was incubated with 1–2 MOI of hOC43, SARS-CoV-2-zsGreen, or SARS-CoV-2-delta for 30 minutes at 33°C (OC43) or 37°C (SARS-CoV-2). Media was aspirated from cells and ACE2/virus complex was added to cells and incubated for 30–60 minutes, followed by the addition of complete media containing 10% FBS and 1× NEAA. Cells were incubated for 7–24 hours. Cells were fixed with a final concentration of 1 or 4% PFA and run on a flow cytometer or antibody stained followed by flow cytometry.

### Antibody staining

After initial fixation, cells were incubated with BD Cytofix solution and permeabilized with BD Cytoperm solution (BD Cytofix/Cytoperm Kit). Cells infected with OC43 were stained with anti-Coronavirus Group Antigen Antibody, nucleoprotein of OC-43, clone 542–7D (Millipore) at a dilution of 1:500, and goat anti-mouse Alexa Fluor 488 secondary at a dilution of 1:1,000. Cells infected with the SARS-CoV-2 delta variant were stained with SARS-CoV/SARS-CoV-2 Nucleocapsid Antibody at a dilution of 1:1,000 (Sino Biological) and goat anti-mouse Alexa Fluor 488 secondary at a dilution of 1:1,000 (Life Technologies). Stained cells were analyzed on a Stratedigm S1000EON bench flow cytometer.

### Cell viability and toxicity assays

A threefold dilution series of sACE2^WT^ and sACE2^T371W^ starting at 333 µM was diluted in media containing DMEM, 10% FBS and, 1× non-essential amino acids. 10,000 Huh7.5-mutACE2 or A549-TMPRSS2-ACE2 cells were added to respective wells of each dilution and incubated for 48 hours. Cell viability was determined by CellTiter-Glo assay per the manufacturer’s instructions (Promega). The background signal was subtracted, and the percent viability was determined by dividing the sample value by the corresponding untreated sample. Cell toxicity was determined by LDH-Glo Cytotoxicity assay per the manufacturer’s instructions (Promega). The background signal was subtracted, and all negative values were converted to zero.

### Mice

Male and female K18-hACE2 mice (Jackson Laboratory) 6–8 weeks were used for these studies. Mice were housed according to NIH guidelines for housing and care in a Biosafety Level 3 animal laboratory. All procedures were approved by the Institutional Animal Care and Use Committee (protocol number 2020-102987) of the University of Texas Southwestern.

### *In vivo* infections and intranasal and nebulizer treatments

K18-hACE2 mice were anesthetized with ketamine/xylazine (80/6 mg/kg) and treated with soluble ACE2^T371W^ (sACE2 ^T371W^; 8.5–12 mg/mL) or buffer control using a nebulizer (Animal holder nebulizer delivery system, Kent Scientific). Each mouse was placed in the holder and 125 μL of sACE2^T371W^ or buffer was added to the nebulizer and nebulized at 0.2 L/min. After the vaper was cleared, each mouse was removed from the restraint and the process was repeated for each additional mouse. At 30 minutes post-treatment, mice were intranasally (IN) infected with 1 × 10^3^ PFU of SARS-CoV-2 P.1 variant suspended in 30 µl of phosphate-buffered saline (PBS). For low-dose infections, K18-hACE2 mice were anesthetized with ketamine/xylazine (80/6 mg/kg) and intranasally (IN) infected with 1 × 10^2^ PFU of SARS-CoV-2 P.1 variant suspended in 30 µl of phosphate-buffered saline (PBS) and treated 20 µl IN with soluble ACE2^T371W^ (sACE2^T371W^; 11.6–12 mg/mL) or control 30 min either before or after infection or given 1dpi, 2dpi, or combination of 1dpi and 2dpi. All mice were monitored daily for weight and mortality. Animals that lost more than 20% of their original body weight or appeared lethargic, hunched, or unable to obtain food were euthanized per Institutional Animal Care and Use Committee guidelines.

For quantifying the SARS-CoV-2 viral load, whole lungs were homogenized in 600 μl 2% FBS (Gibco)/MEM (Gibco) with stainless steel beads (NextAdvance) in a Bullet Blender Pro (NextAdvance). Lung tissue was weighed before homogenization. Tissue homogenates were clarified of debris by centrifugation at 4°C at 800 *× g* for 5 minutes, and aliquots for plaque assay were frozen at −80°C. Plaque assays were performed as described above.

## AFTER EPUB

*[This article was published on 21 May 2024 with an error in Matthew B. McDougal's given name. The name was corrected in the current version, posted on 3 June 2024.]*

## SUPPLEMENTAL MATERIAL

Supplemental figures mbio.00768-24-s0001.pdf Fig. S1 to S3.

ASM does not own the copyrights to Supplemental Material that may be linked to, or accessed through, an article. The authors have granted ASM a non-exclusive, world-wide license to publish the Supplemental Material files. Please contact the corresponding author directly for reuse.
