# Supplementary material for: Development of a mutant aerosolized ACE2 that neutralizes SARS-CoV-2 in vivo
Source: mBio. 2024 May 21;15(6):e00768-24. doi: 10.1128/mbio.00768-24 (PMC11237572; doi:10.1128/mbio.00768-24)
Supplement: Supplemental figures — Fig. S1 to S3. [file mbio.00768-24-s0001.pdf]

Figure S1.

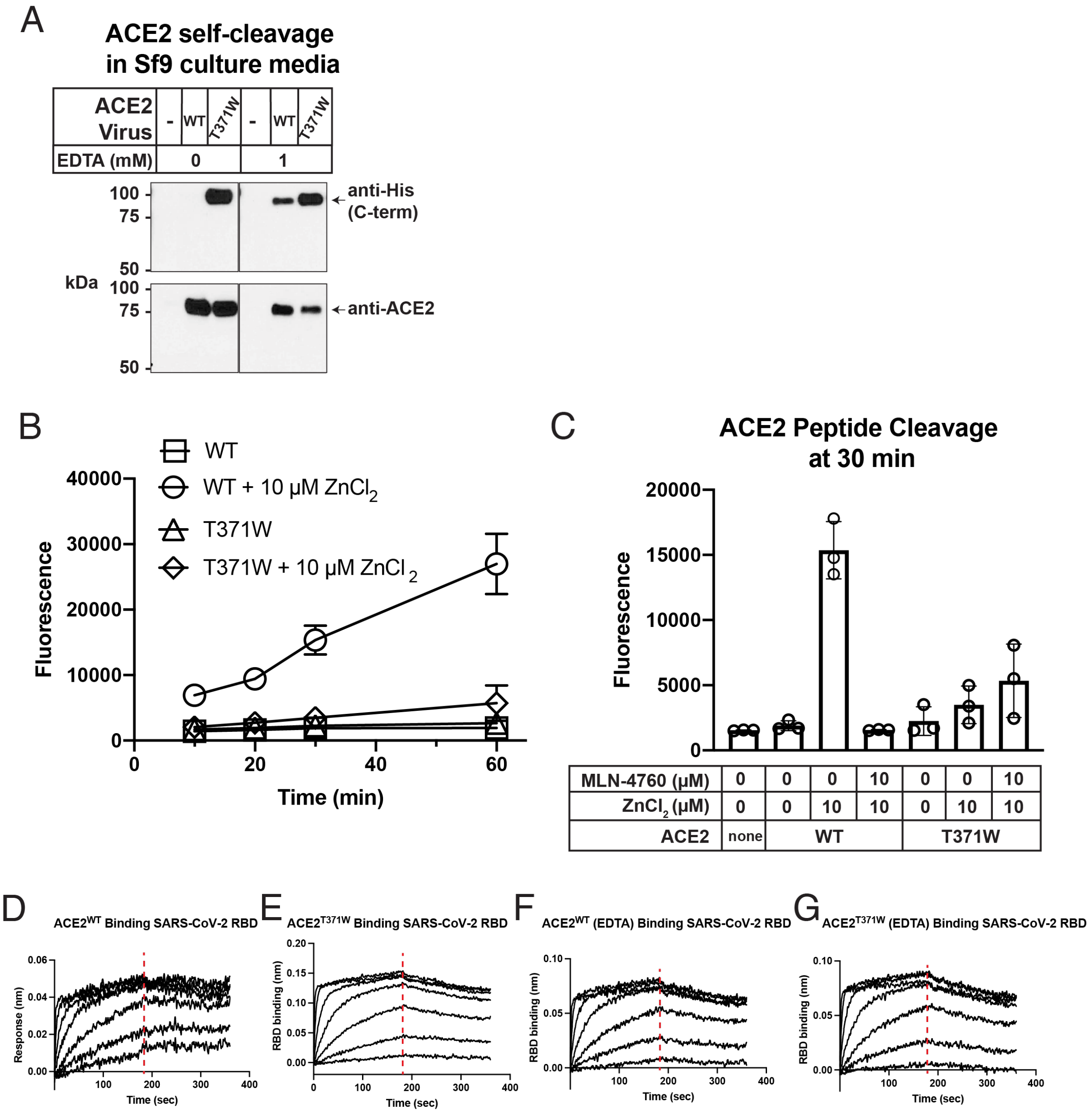
A
ACE2 self-cleavage in Sf9 culture media
ACE2 Virus
-
WT
T371W
-
WT
T371W
EDTA (mM)
0
1
100
75
50
kDa
100
75
50
anti-His (C-term)
anti-ACE2
B
WT
WT + 10 μM ZnCl2
T371W
T371W + 10 μM ZnCl 2
40000
30000
20000
10000
0
Fluorescence
0
20
40
60
Time (min)
C
ACE2 Peptide Cleavage at 30 min
20000
15000
10000
5000
0
Fluorescence
MLN-4760 (μM) 0 0 0 10 0 0 10
ZnCl2 (μM) 0 0 10 10 0 10 10
ACE2 none WT T371W
D
ACE2WT Binding SARS-CoV-2 RBD
Response (nm)
0.06
0.04
0.02
0.00
100
200
300
400
Time (sec)
E
ACE2T371W Binding SARS-CoV-2 RBD
RBD binding (nm)
0.20
0.15
0.10
0.05
0.00
0
100
200
300
400
Time (sec)
F
ACE2WT (EDTA) Binding SARS-CoV-2 RBD
RBD binding (nm)
0.10
0.05
0.00
100
200
300
400
Time (sec)
G
ACE2T371W (EDTA) Binding SARS-CoV-2 RBD
RBD binding (nm)
0.10
0.05
0.00
100
200
300
400
Time (sec)

Figure S1. Characterization of ACE2^{T371W} enzymatic activity

A) ACE2 self-cleavage in insect cell culture media. Sf9 cells were infected with the indicated virus for 48 hrs. Conditioned supernatants were collected and subjected to immunoblot analysis with either anti-ACE2 or anti-His antibodies.

B) Peptide cleavage by ACE2 ectodomains monitored through Mca fluorescence. Proteins were treated with $ZnCl_2$ as indicated and fluorescence was recorded at the indicated time points.

C) ACE2 peptide cleavage at 30 minutes monitored through Mca fluorescence in the presence or absence of ZnCl2 and MLN-4760 inhibitor, as indicated.

D) Sensorgram traces showing binding between ACE2^{WT} ectodomains expressed without EDTA binding SARS-CoV-2 RBD domains. SARS-CoV-2 RBD domains were titrated from 1000 nM to ~1.4 nM in a three-fold dilution series. Dashed red line at t = 180 seconds indicates the switching of the biosensors from with RBD to the buffer-only washing well.

E) Sensorgram traces showing binding between ACE2^{T371} ectodomains expressed without EDTA binding SARS-CoV-2 RBD domains. SARS-CoV-2 RBD domains were titrated from 1000 nM to ~1.4 nM in a three-fold dilution series. Dashed red line at t = 180 seconds indicates the switching of the biosensors from with RBD to the buffer-only washing well.

F) Sensorgram traces showing binding between ACE2^{WT} ectodomains expressed with 1mM EDTA binding SARS-CoV-2 RBD domains. SARS-CoV-2 RBD domains were titrated from 1000 nM to ~1.4 nM in a three-fold dilution series.

Dashed red line at t = 180 seconds indicates the switching of the biosensors from with RBD to the buffer-only washing well.

G) Sensorgram traces showing binding between ACE2^{T371W} ectodomains expressed with 1mM EDTA binding SARS-CoV-2 RBD domains. SARS-CoV-2 RBD domains were titrated from 1000 nM to ~1.4 nM in a three-fold dilution series. Dashed red line at t = 180 seconds indicates the switching of the biosensors from with RBD to the buffer-only washing well.

Figure S2

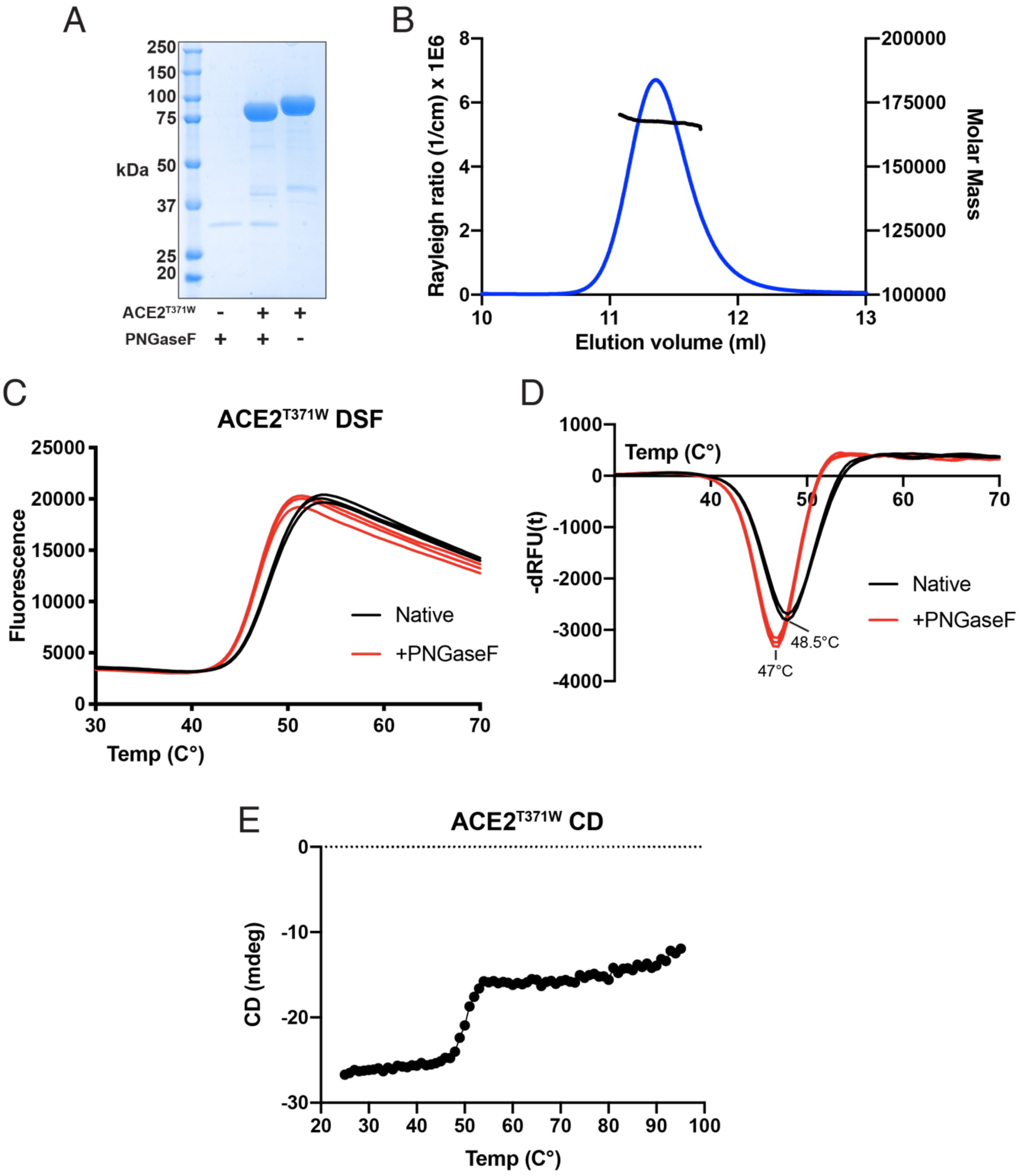

A
250
150
100
75
kDa
50
37
25
20
ACE2T371W - + +
PNGaseF + + -
B
Rayleigh ratio (1/cm) x 1E6
Molar Mass
Elution volume (ml)
200000
175000
150000
125000
100000
C
ACE2T371W DSF
Fluorescence
Temp (C°)
Native
+PNGaseF
D
Temp (C°)
-dRFU(t)
Native
+PNGaseF
48.5°C
47°C
E
ACE2T371W CD
CD (mdeg)
Temp (C°)

Figure S2. Solution characterization of ACE2^{T371W} ectodomains

A) Coomassie-stained SDS-PAGE analysis of ACE2^{T371W} ectodomains subjected to PNGaseF treatment.
B) SEC-MALS analysis of purified ACE2^{T371W} ectodomains. Rayleigh scattering is shown in blue trace and the calculated molar mass is show in the black trace.
C) Fluorescent traces from DSF experiment with native and PNGaseF-digested ACE2^{T371W}.
D) Derivative curves from the fluorescent traces shown in (C).
E) CD melt trace for signal at 222 nm for ACE2^{T371W}.

Figure S3

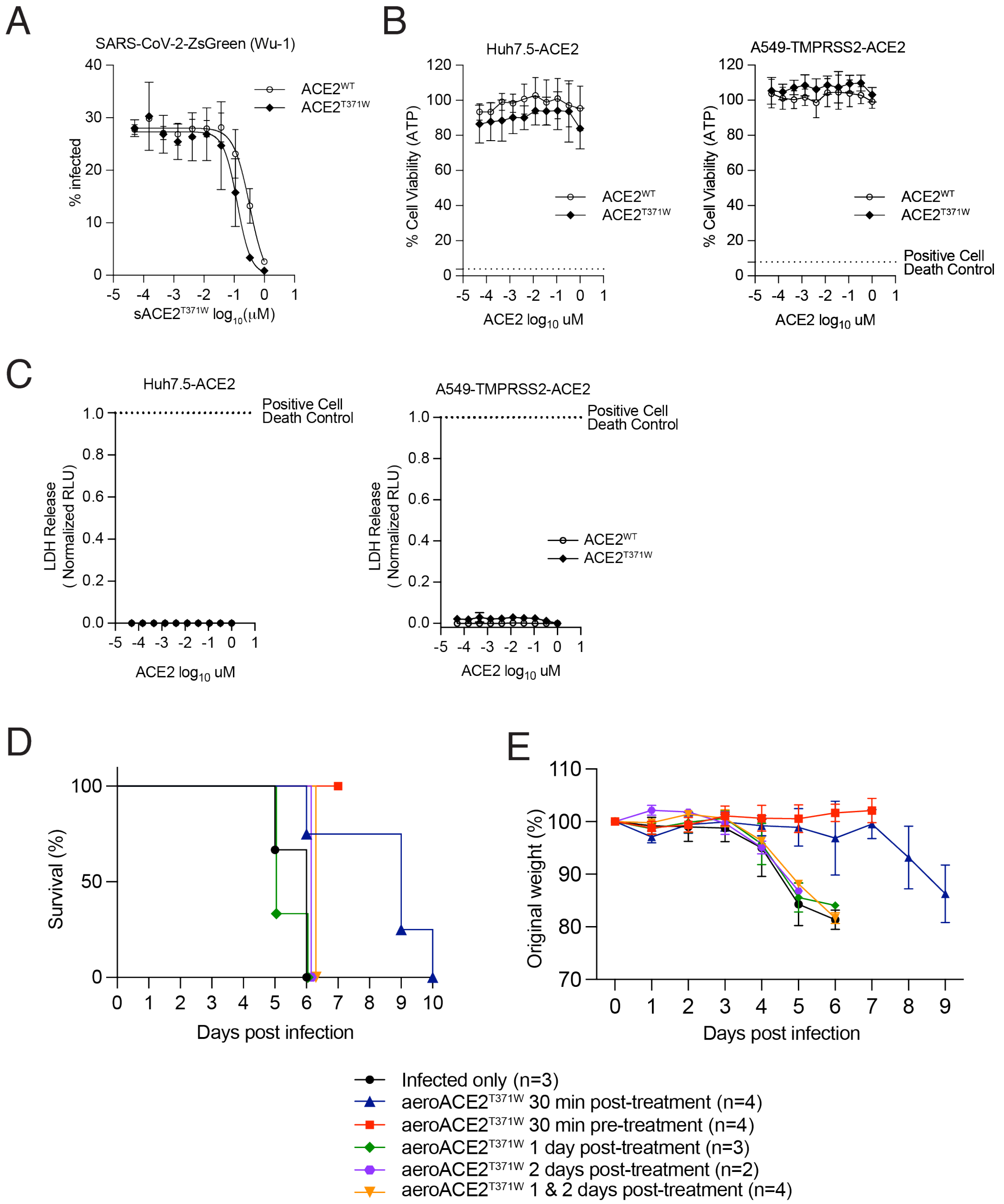
A
SARS-CoV-2-ZsGreen (Wu-1)
% infected
sACE2^{T371W} log$_{10}$(μM)
ACE2^{WT}
ACE2^{T371W}
B
Huh7.5-ACE2
A549-TMPRSS2-ACE2
% Cell Viability (ATP)
ACE2 log$_{10}$ uM
Positive Cell Death Control
C
LDH Release (Normalized RLU)
D
Survival (%)
Days post infection
E
Original weight (%)
Infected only (n=3)
aeroACE2^{T371W} 30 min post-treatment (n=4)
aeroACE2^{T371W} 30 min pre-treatment (n=4)
aeroACE2^{T371W} 1 day post-treatment (n=3)
aeroACE2^{T371W} 2 days post-treatment (n=2)
aeroACE2^{T371W} 1 & 2 days post-treatment (n=4)

Figure S3. Intranasal pretreatment of sACE2 inhibits SARS-CoV-2 replication.

A) Infectivity of SARS-CoV-2-Wu-1-zsGreen in the presence of varying concentrations of sACE2^{T371W} or sACE2^{WT}. sACE2^{T371W} or sACE2WT was incubated with 1-2 MOI of SARS-CoV-2 variants for 30 minutes at 37°C. Each ACE2/virus complex was transferred to 100,000 A549-TMPRSS2-ACE2 cells, followed by incubation at 37°C. At 7 hrs post-infection, cells were fixed with 4% PFA and viral infectivity was quantified by flow cytometry. n=2 biological replicates.

B) Cell viability of Huh7.5-ACE2 and A549-TMPRSS2-ACE2 cells treated with varying concentrations of sACE2^{T371W} or sACE2^{WT}. sACE2^{T371W} or sACE2^{WT} were added to 10,000 Huh7.5mutACE2 and A549-TMPRSS2-ACE2 cells. 48h hr later, ATP levels in treated cells were quantified by Cell Titer Glo assay. n=3 biological replicates.

C) Cell toxicity of Huh7.5-ACE2 and A549-TMPRSS2-ACE2 cells treated with varying concentrations of sACE2T371W or sACE2WT. sACE2^{T371W} or sACE2^{WT} were added to 10,000 Huh7.5mutACE2 and A549-TMPRSS2-ACE2 cells. 48h hr later, LDH release into cell supernatants was quantified by Cell Titer Glo assay. n=3 biological replicates.

D) and E) Survival and daily weight of K18-hACE2 mice treated intranasally either 30min prior to infection with SARS-CoV-2 P.1 or 30min, 1 day, 2 day, or 1+2 day post infection or infected alone. Infected only and 30min prior to infection treatment were given 60,000 PFU of SARS-CoV-2 P.1. All other groups were infected with 100 PFU of SARS-CoV-2 P.1. All female mice were used for these experiments.
